# Impact of a Blended-Learning Rapid Sequence Intubation Training Program on Emergency Physician Confidence: A Single-Center Program Evaluation

**DOI:** 10.2147/OAEM.S574489

**Published:** 2026-02-24

**Authors:** Shabbir Ahmad, Zainab Ahmed Alani, Yavuz Yigit, Shahzad Anjum, Khalid Bashir

**Affiliations:** 1 Emergency Department, Hamad General Hospital, Doha, Qatar; 2 University of Glasgow School of Medicine, Glasgow, UK; 3 Clinical Department, College of Medicine, QU Health, Qatar University, Doha, Qatar

**Keywords:** rapid sequence intubation, blended learning, simulation-based education, emergency medicine training, program evaluation, physician confidence

## Abstract

**Background:**

Rapid Sequence Intubation (RSI) is a high-risk, time-critical procedure in emergency medicine. Standardizing its practice across a multinational workforce with diverse training backgrounds remains a persistent educational challenge. This mixed-methods program evaluation assessed a blended-learning RSI program’s impact on self-reported physician confidence and perceived educational value within a tertiary emergency care setting.

**Methods:**

A retrospective evaluation was conducted for a blended-learning RSI curriculum delivered between 2020 and 2022. The program combined asynchronous online modules with a 4-hour, small-group simulation-based practical session. A voluntary, anonymous post-program survey was administered in 2025 following IRB approval. Outcomes included retrospective pre-post self-reported confidence (5-point Likert scale), satisfaction with program components, and qualitative feedback. No objective performance measures were assessed.

**Results:**

Forty-five of 141 eligible physicians completed the survey (adjusted response rate 42%). Self-reported low confidence decreased from 77.8% pre-course to 6.6% post-course, while high confidence increased from 22.2% to 93.4% (Cohen’s d = 2.1). Improvements were consistent across professional grades. The simulation-based session received the highest satisfaction rating (mean 4.3/5). Qualitative analysis highlighted the value of realistic simulation, structured debriefing, and standardized checklists. Suggested improvements included more complex scenarios and enhanced online interactivity.

**Conclusion:**

Participation in a blended-learning RSI program was associated with substantial improvement in self-reported physician confidence and high satisfaction among a multinational emergency medicine workforce. These findings support the feasibility and perceived value of this educational approach for high-risk procedural training in similar settings, though objective competence was not evaluated.

## Introduction

Rapid Sequence Intubation (RSI) represents a fundamental and high-stakes component of emergency airway management, designed to facilitate rapid orotracheal intubation while minimizing aspiration risk in critically ill patients.^1^ Its optimal execution requires precise coordination of pharmacologic induction, neuromuscular blockade, pre-oxygenation, and contingency planning to prevent complications such as hypoxemia and hemodynamic collapse.^2^ Despite its established role, RSI remains associated with significant morbidity, particularly within the dynamic and high-pressure environment of the Emergency Department (ED).^3^,^4^

This risk is amplified in large tertiary care centers employing a multinational physician workforce. Variability in prior training backgrounds—spanning different healthcare systems, protocols, and exposure levels—can lead to inconsistent procedural practices, potentially compromising patient safety and standardization of care.^5^,^6^ The COVID-19 pandemic further accentuated the need for rigorous, standardized airway training, as RSI became an aerosol-generating procedure requiring strict infection control measures, flawless team coordination, and rapid, decisive action under heightened stress.^7^,^8^

Simulation-based education has emerged as a cornerstone for training high-risk, low-frequency procedures in emergency medicine, providing a safe environment for deliberate practice, error management, and team skill development.^9^,^10^ Blended-learning models, which integrate asynchronous online instruction with hands-on simulation, optimize this approach by allowing flexible knowledge acquisition followed by focused experiential learning.^11^,^12^ While such methods have demonstrated efficacy for a range of procedural skills, evidence evaluating structured, blended RSI training for multinational ED teams remains limited.^6^,^13^

To address this gap, the present study evaluates a mandatory blended-learning RSI program implemented in a large academic ED in Qatar. The program was designed to standardize procedural knowledge, promote a shared mental model among a diverse physician cohort, and enhance confidence in performing RSI safely and efficiently. The primary objective was to assess changes in participants’ self-reported confidence, while secondary objectives included evaluating satisfaction with individual program components and capturing qualitative feedback to guide curriculum refinement.

Although the evaluation did not include objective measures of procedural competence or patient outcomes, assessing perceived confidence and satisfaction provides valuable insight into learners’ preparedness and the perceived educational value of the program. Understanding these dimensions is critical for planning effective procedural training in complex, multinational emergency care settings. By evaluating confidence and satisfaction, this study contributes to the growing evidence supporting blended-learning approaches for high-risk procedural education, particularly within heterogeneous clinical teams. Moreover, the findings may inform the design of scalable, contextually appropriate training interventions that enhance procedural readiness, team coordination, and overall patient safety in diverse ED environments.

## Methods

### Study Design and Setting

This study was a retrospective, single-center, mixed-methods program evaluation conducted within the Emergency Department (ED) of Hamad General Hospital, the principal tertiary academic center in Qatar and an ACGME-I-accredited training institution. The evaluation focused on a mandatory Rapid Sequence Intubation (RSI) training program delivered between January 2020 and December 2022. The post-program evaluation survey was administered in 2025 to assess participants’ perceptions and self-reported confidence (Appendix 1). The study employed a descriptive design and did not include a control or comparator group, reflecting the real-world implementation of a mandatory educational intervention. This design allowed the research team to evaluate program feasibility, learner satisfaction, and self-perceived educational impact in a heterogeneous, multinational clinical workforce.

### Participants

Inclusion criteria comprised all ED physicians who were credentialed to perform RSI as part of their clinical duties and who had completed the entire mandatory training program (both online and in-person components). Exclusion criteria were applied to any physician who had not completed both components of the curriculum. For the voluntary evaluation survey, eligibility was further limited to those who remained employed at the institution at the time of survey distribution (2025). The study cohort included attending physicians (consultants), fellows, and residents, representing a broad spectrum of international training backgrounds. This heterogeneity mirrored the typical composition of the ED workforce and provided a unique opportunity to examine the perceived effectiveness of standardized procedural training across clinicians with diverse prior experiences. Participation in the survey was voluntary, and respondents were assured of anonymity to encourage candid feedback.

### Curriculum Design and Educational Framework

The RSI curriculum was developed based on established adult learning and experiential learning principles,^14^ emphasizing learner-centered instruction, deliberate practice, and structured reflective feedback. The blended-learning design combined asynchronous theoretical instruction with interactive, hands-on simulation to optimize knowledge acquisition and skill consolidation.

### Blended-Learning Components

1. Asynchronous Online Modules (11.5 hours): Delivered via the hospital’s learning management system, these modules covered RSI fundamentals, institutional standard operating procedures, pharmacology and safe sedation practices, a 5-hour Harvard online mechanical ventilation course, COVID-19-specific cardiac arrest management, and human factors and team communication strategies. The online content allowed learners to acquire knowledge flexibly while establishing a foundation for subsequent practical training. Following each module, formative multiple-choice questions (MCQs) and short-answer questions (SAQs) were administered online to reinforce key concepts and provide immediate, self-directed feedback. Completion of these formative assessments was a mandatory requirement for fulfilling the online module component; however, the individual MCQ/SAQ responses were not collected or analyzed as part of this program evaluation.
2. In-Person Practical Session (4 hours): To ensure high-quality, interactive learning, sessions were conducted in small groups of 4–6 participants. Each session was facilitated by **t**wo board-certified emergency physician instructors, each with formal training in simulation-based education and extensive clinical expertise in airway management. The practical component included:
  - A 1-hour airway skills workshop focusing on equipment preparation, drug handling, and safety checks.
  - A 3-hour simulation-based course comprising four progressive clinical scenarios utilizing medium-fidelity manikins. Scenarios emphasized technical skills, standardized checklist application, and non-technical skills such as situational awareness, team coordination, and communication. Each scenario concluded with a structured, facilitator-led debriefing to reinforce learning points and promote reflective practice.

### Outcome Measures and Data Collection

The evaluation was guided by Kirkpatrick’s Levels 1 (Reaction) and 2 (Learning).^15^ Data were collected through a confidential, online post-program survey distributed in 2025.

- Primary Outcome: Change in self-reported confidence in performing RSI, retrospectively rated on a 5-point Likert scale (1 = Very Low, 5 = Very High) for pre- and post-course states.
- Secondary Outcomes: Included satisfaction with individual program components (online modules, simulation sessions, structured debriefing, and small-group format), overall perceived educational value, likelihood to recommend the program, and qualitative feedback on program strengths and areas for improvement collected via open-ended questions.

### Data Analysis

A convergent mixed-methods approach was employed.

- Quantitative Analysis: Descriptive statistics were calculated using IBM SPSS Statistics (v28.0) and reported as frequencies, percentages, means, and standard deviations. The magnitude of confidence change was quantified using Cohen’s d and 95% confidence intervals were reported for satisfaction scores. Inferential testing was not the primary objective given the descriptive nature of the program evaluation.
- Qualitative Analysis:** **Inductive thematic analysis of open-ended responses was conducted by two independent investigators who coded responses, identified emergent themes, and resolved discrepancies through discussion to ensure interpretive consistency.

### Ethical Considerations

The study was reviewed and approved by the Hamad Medical Corporation Institutional Review Board (MRC-01-24-472) in 2024 when the decision was made to publish the evaluation findings, and was conducted in accordance with the Declaration of Helsinki. The RSI training itself was a mandatory clinical safety initiative, whereas participation in the evaluation survey was entirely voluntary. Survey completion implied informed consent, and no adverse consequences affected employment or credentialing. To mitigate potential non-response and selection bias, the survey was distributed with multiple reminders and explicit assurance of anonymity. The study acknowledges the potential for social desirability bias in self-reported data.

## Results

### Participant Characteristics and Response

Of the 141 physicians who completed the RSI training program, 45 responded to the post-program survey, yielding a raw response rate of 31.9%. After excluding 22 physicians who had left the institution during the interim, the adjusted response rate was 42% (45/119). Respondents included attending physicians (46.7%, n=21), fellows (22.2%, n=10), and residents (31.1%, n=14), reflecting a representative distribution across professional grades. The cohort was predominantly male (91.1%), consistent with the departmental demographics at the time, and reported postgraduate training spanning over 15 countries. This diversity underscores the multinational composition of the workforce and highlights the need for standardized procedural training to ensure consistent practice across clinicians with varied prior exposure and training backgrounds.

### Self-Reported Confidence in RSI Performance

A substantial positive shift in self-reported confidence was observed following completion of the blended-learning program (Table 1). Pre-course, 77.8% of respondents (n=35) reported low confidence (Likert score 1–2), which decreased to 6.6% (n=3) post-course. Conversely, the proportion of participants reporting high confidence (score 4–5) increased from 22.2% (n=10) to 93.4% (n=42). The calculated effect size was large (Cohen’s *d* = 2.1), indicating a marked improvement in perceived readiness to perform RSI. These gains were consistent across all professional grades, with non-board-certified trainees showing the largest relative improvement. The observed increase in confidence is clinically relevant, suggesting that the blended-learning program effectively enhanced participants’ perceived ability to perform a high-risk procedure in a complex emergency setting.

**Table 1:** Retrospective Pre- and Post-Course Self-Reported Confidence (N=45)

Confidence Level	Pre-Course, n (%)	Post-Course, n (%)
Low (Score 1–2)	35 (77.8)	**3 (6.6)**
Neutral (Score 3)	0 (0.0)	0 (0.0)
High (Score 4–5)	10 (22.2)	**42 (93.4)**

**Notes**: Likert Scale: 1 = Very Low Confidence, 5 = Very High Confidence. Bold values indicate the primary outcome of interest: post-course high confidence.

### Satisfaction with Program Components

Overall satisfaction with the program was high, with a mean score of 4.2 (SD 0.7) across all components (Table 2). Among specific elements, the simulation-based practical session received the highest rating (mean 4.3, SD 0.6), followed closely by structured debriefing (mean 4.2, SD 0.7) and small-group learning format (mean 4.1, SD 0.8). Faculty expertise and feedback were highly rated (mean 4.4, SD 0.5), reflecting the perceived value of instructor-led guidance. The asynchronous online modules received slightly lower, yet positive, ratings (mean 3.8, SD 0.9), with qualitative feedback suggesting the desire for more interactive content. The higher satisfaction scores for simulation and debriefing likely reflect the benefits of experiential, hands-on learning and immediate feedback, which are critical for skill acquisition in high-risk, low-frequency procedures.

**Table 2:** Mean Participant Satisfaction Scores for Key Components of the Simulation-Based Learning Program

Program Component	Mean Score (SD)	95% Confidence Interval
Simulation-Based Practical Session	**4.3 (0.6)**	4.1–4.5
Structured Debriefing	4.2 (0.7)	4.0–4.4
Small-Group Learning Format	4.1 (0.8)	3.9–4.3
Faculty Expertise & Feedback	**4.4 (0.5)**	4.3–4.5
Asynchronous Online Modules	3.8 (0.9)	3.5–4.1
Overall Program Satisfaction	4.2 (0.7)	4.0–4.4

**Notes**: Bold values indicate the highest-rated program components. SD = Standard Deviation.

### Qualitative Feedback

Thematic analysis of open-ended responses identified three key themes. First, participants emphasized the educational value of experiential learning, citing realistic simulation and structured debriefing as central to reinforcing procedural steps, improving team communication, and reducing anxiety during high-stakes scenarios. Second, respondents reported enhanced perceived preparedness, including greater organization, systematic approach, and familiarity with RSI checklists, pharmacology, and complication management. Third, participants provided constructive suggestions for program improvement, including incorporation of more complex or atypical airway scenarios, expansion to interprofessional training for nursing and respiratory therapy staff, and increased interactivity within online modules. These qualitative insights complement quantitative findings, providing actionable recommendations to enhance future curriculum iterations and further standardize airway management training across a multinational workforce.

## Discussion

### Principal Findings

This program evaluation demonstrated that a structured, blended-learning RSI curriculum was associated with substantial, self-reported improvements in physician confidence within a multinational Emergency Department. The large effect size (Cohen’s *d* = 2.1) and consistently high satisfaction ratings, particularly for simulation-based sessions and structured debriefing, indicate that the program was both acceptable and subjectively effective from the participants’ perspective. Qualitative feedback reinforced these findings, highlighting the perceived value of hands-on, team-based experiential learning in consolidating procedural knowledge, enhancing preparedness, and fostering systematic approaches to high-risk airway management. Participants reported that the program improved their organization, situational awareness, and confidence in managing complex airway scenarios. Collectively, these data suggest that a blended-learning model can effectively enhance learners’ confidence—a critical precursor to safe and effective clinical practice in high-acuity settings.

### Interpretation in Context

The substantial confidence gains align with prior literature demonstrating the efficacy of simulation-based and blended-learning approaches for procedural skill acquisition in emergency medicine.^9^,^11^,^12^ Standardized curricula have been shown to reduce variability in procedural performance, particularly in heterogeneous groups comprising physicians from diverse international training backgrounds.^5^,^6^ This is particularly relevant in internationally staffed EDs, where inconsistent prior exposure to RSI protocols can compromise team performance and patient safety.

High valuation of simulation and structured debriefing supports experiential learning theory, which emphasizes reflection on concrete experience as essential for skill consolidation and development of adaptive expertise.^14^ Participants’ feedback indicating that structured debriefing transformed practical exercises into effective learning underscores its importance in procedural training. Furthermore, requests for more complex scenarios and interprofessional inclusion, such as integration of nursing and respiratory therapy staff, reflect a desire for realistic, team-based training that mirrors clinical practice. These insights align with evidence that interprofessional simulation improves team communication, collaboration, and patient outcomes,^16^ emphasizing the relevance of expanding training beyond physicians alone.

### Limitations

Our study has several limitations. First, this evaluation focused on self-reported confidence rather than objective performance metrics. While confidence does not equate to verified clinical competence, it represents a relevant educational construct aligned with perceived readiness for high-risk procedures and is commonly used in program evaluations at Kirkpatrick Level 2.

Second, confidence was assessed using a retrospective pre–post survey administered after course completion. This approach, frequently employed in educational research to account for response-shift effects, may introduce recall bias; accordingly, observed changes likely reflect the combined influence of structured training and subsequent clinical exposure rather than a single isolated effect.Third, the adjusted response rate of 42% reflects the transient nature of a multinational emergency medicine workforce, with a substantial proportion of the original cohort relocating internationally during the follow-up period. While selection bias is possible, the demographic distribution of respondents across professional grades and the internal consistency of quantitative and qualitative findings support the credibility of the observed trends.Fourth, the absence of a control group limits causal inference, consistent with the descriptive intent of this program evaluation. The study was not designed to isolate the effect of the intervention from concurrent clinical experience or parallel educational activities, nor to assess long-term skill retention or patient-level outcomes.Despite these considerations, the convergence of quantitative confidence gains and qualitative feedback demonstrates meaningful perceived educational impact. Participants consistently described enhanced preparedness, structured workflow, and improved team coordination, supporting the feasibility and educational value of a mandatory blended-learning RSI program implemented at scale within a complex, multinational emergency department setting.

### Implications for Practice and Future Research

Despite limitations, this evaluation demonstrates the feasibility, acceptability, and perceived educational value of blended-learning RSI training in a real-world, multinational ED setting. The program provides a replicable framework for standardizing high-risk procedural training across diverse clinician backgrounds, fostering shared mental models, effective team coordination, and confidence in managing critically ill patients.

Future research should focus on strengthening the evidence base through prospective, controlled study designs using validated objective assessment tools and patient-centered outcomes, such as first-pass success or complication rates. Longitudinal follow-up would enable evaluation of skill retention and long-term clinical impact. Multi-center studies could assess generalizability across healthcare systems and diverse learner populations. Expanding interprofessional components to include nurses, respiratory therapists, and other team members may further enhance team performance and patient safety, reflecting the growing emphasis on collaborative, patient-centered care in emergency medicine.

## Conclusion

This single-center program evaluation found that a blended-learning RSI training program was associated with significant improvements in self-reported physician confidence and high participant satisfaction among a multinational emergency medicine workforce. While objective procedural competence was not assessed, the findings support the feasibility, acceptability, and perceived educational value of this blended-learning model for standardizing high-risk procedural training. These results are particularly relevant for institutions with transient or internationally trained teams and provide a foundation for ongoing curriculum refinement and future research incorporating objective performance assessment.
